# Cell Transplantation for Spinal Cord Injury: Tumorigenicity of Induced Pluripotent Stem Cell-Derived Neural Stem/Progenitor Cells

**DOI:** 10.1155/2018/5653787

**Published:** 2018-02-04

**Authors:** Junhao Deng, Yiling Zhang, Yong Xie, Licheng Zhang, Peifu Tang

**Affiliations:** Department of Orthopaedics, Chinese PLA General Hospital, No. 28 Fuxin Road, Beijing 100853, China

## Abstract

Spinal cord injury (SCI) is an intractable and worldwide difficult medical challenge with limited treatments. Neural stem/progenitor cell (NS/PC) transplantation derived from fetal tissues or embryonic stem cells (ESCs) has demonstrated therapeutic effects via replacement of lost neurons and severed axons and creation of permissive microenvironment to promote repair of spinal cord and axon regeneration but causes ethnical concerns and immunological rejections as well. Thus, the implementation of induced pluripotent stem cells (iPSCs), which can be generated from adult somatic cells and differentiated into NS/PCs, provides an effective alternation in the treatment of SCI. However, as researches further deepen, there is accumulating evidence that the use of iPSC-derived NS/PCs shows mounting concerns of safety, especially the tumorigenicity. This review discusses the tumorigenicity of iPSC-derived NS/PCs focusing on the two different routes of tumorigenicity (teratomas and true tumors) and underlying mechanisms behind them, as well as possible solutions to circumvent them.

## 1. Background

Spinal cord injury is a devastating neurological condition, which results in the disruption of signals between the brain and body yielding severe physical, psychological, and social dysfunction [1, 2]. Patients who have suffered a SCI not only become increasingly dependent on others for daily life but are more likely to die prematurely and are at risk for social exclusion [1, 2]. What is worse is that, due to the complex pathophysiological processes, significant treatment for SCI has progressed slowly.

Originally, glucocorticoid drugs like methylprednisolone were regarded as the classic therapeutic treatment for SCI [3], as they had been found to stabilize the plasma membrane of damaged cells by inhibiting lipid peroxidation and hydrolysis [3]. However, their application gradually became controversial because they had serious side effects like mounting vulnerability to acute corticosteroid myopathy or serious infection [4, 5]. Other clinical approaches to SCI included early surgical interventions [6] and alternative pharmacological therapy (e.g., GM-1 [7] and thyrotropin-releasing hormone [8]). However, these methods either had their own side effects or demonstrated weakly therapeutic efficacy.

Recent progress in cell transplantation has opened up new opportunities to understand and treat SCI. Among the several types of candidate cells, NS/PC holds great therapeutic potential for SCI, as it can replace the lost neurons and glia as well as create a growth-promoting environment [9]. Nevertheless, the acquisition of NS/PCs can be a difficult task since they are usually located deep in the brain so their isolation is a highly invasive procedure. To bypass this problem, people have also used ESCs from which they can generate sufficient NS/PCs. Indeed, ESC-derived NS/PCs were initially reported to have optimistic effects on SCI [10, 11]. Unfortunately, the application of ESC-based strategy, accompanied by immune rejections and ethical concerns [12], was less likely to be transformed into clinical practice. Subsequently, the advent of iPSCs appears to signal the future of stem cell treatments for SCI. However, while the therapeutic effects of iPSCs on SCI have been discussed by many studies, the side effects are rarely mentioned and talked over exclusively, especially the tumorigenicity of iPSCs. In this paper, we briefly summarized the application of iPSCs, elucidated the tumorigenicity in detail, and described possible strategies to address it.

## 2. Application of iPSCs in Spinal Cord Injury: An Overview

In 2006, Takahashi and Yamanaka showed that fibroblasts from mouse somatic cells could regain pluripotency after expressing four transcriptional factors [13], thus developing iPSCs. It stands to reason that iPSCs may have the greatest potential for regenerative medicine, because they have abilities to indefinitely self-renew and differentiate into most if not all cell types [13, 14]. Compared to ESCs, autologous iPSCs also circumvent the ethical issues associated with embryonic tissue harvesting and free patients of immunosuppression, which is critical since SCI patients are at high risk for infection [15].

Of late, an increasing number of research groups have applied iPSCs to SCI and achieved interesting results (Table 1). In 2010, Tsuji et al. managed to produce neurospheres from mouse iPSCs and showed that transplantation of these cells promoted functional improvement in mice with SCI [16]. As a proof of principle, they also used human iPSCs (hiPSCs) and demonstrated significant therapeutic effects like the better recovery of motor function, synapse formation between the grafts and hosts, and enhanced axonal regrowth [17]. Kobayashi et al. transplanted hiPSC-derived NS/PCs into a nonhuman primate following cervical SCI and revealed behavioral improvements consistent with rodent studies [18]. Lu et al. reported that not only can the derivatives of iPSCs extend axons over nearly the whole length of the rat CNS [19] but can also form extensive synaptic connections with the host. More recently, several studies have elucidated potential mechanisms underlying behavioral improvement from SCI following transplantation of iPSC derivatives [20, 21]. They speculated that iPSC derivatives exerted their effects on SCI by substitution of lost neural cells, promotion of axonal remyelination, and regrowth as well as tissue sparing through trophic support.

There are also some negative reports on iPSC approaches to SCI. Two reports revealed that despite the ability to differentiate into neural cells [19, 22], iPSC-derived NS/PCs did not show any substantial improvement in function. Besides, it takes a long time to generate and evaluate iPSCs [23], making it unrealistic for individualized iPSC-based therapy because the optimal time for stem cell transplantation is the subacute phase [24]. As a result, either iPSCs would have to be generated from donor tissue, missing out on the major factor that makes them attractive in the first place, or transplanted at more chronic phases of injury [25], which showed a poor result after transplantation into the chronic SCI model. More importantly, like ESCs, there are widely found issues with respect to safety of iPSCs, particularly the possible tumorigenicity [16, 21, 26].

## 3. Characteristics and Underlying Mechanisms of iPSC in Tumorigenicity

**Figure 1:**
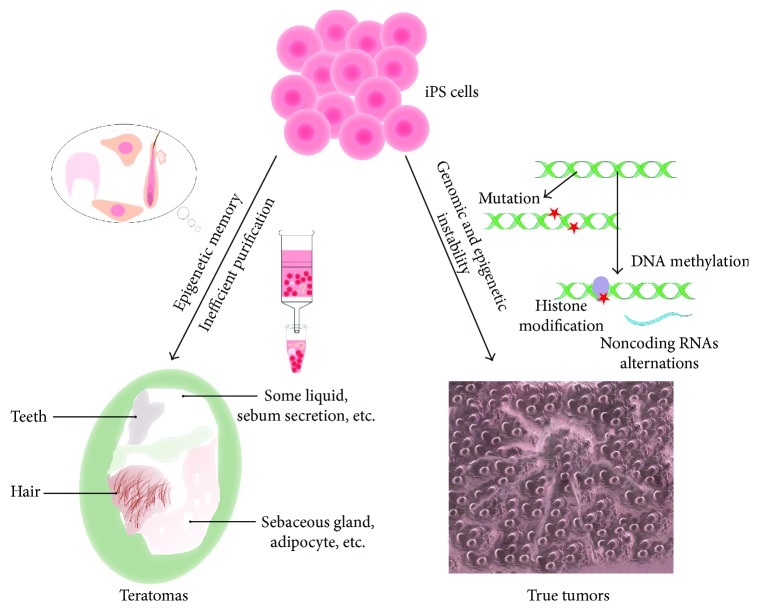
Two distinguished aspects of tumorigenicity and respective potential mechanisms. The tumorigenicity of iPSCs included two parts: one of them is teratoma formation and another is substantial tumor formation. The former is mostly attributed to epigenetic memory as well as inefficient purification, while the latter is ascribed to genomic instability and epigenetic changes.

Tumorigenicity of any stem cell transplants remains a major concern for clinical applications, and there is an urgent need for it to be addressed before translation of iPSC techniques into SCI treatment. From several reports [26, 27], tumorigenicity of iPSCs can be classified into two distinct types: teratoma and true tumors due to their different features and developmental processes, which we will discuss further below (Figure 1).

### 3.1. Teratoma Formation

Teratoma is a relatively common potential risk in grafts of iPSCs especially when individual iPSC clones were preevaluated as unsafe [16, 17, 28]. While the mechanism is not fully understood, most reports share the idea that undifferentiated iPSCs lead to teratoma formation [26, 29]. Teratoma formation requires the ability to escape or silence the immune responses for the purpose of survival in the host. Tumor cells could take effective measures to avoid immune responses by alteration of MHC-I, mutations in Fas or Trail, and so forth [30]. These traits are well shared with undifferentiated iPSCs. Besides, like tumor cells, iPSCs possess a virtually unlimited proliferation potential, by which they are vulnerable to the formation of a cell mass. Therefore, we reasonably postulate that residual-undifferentiated cells contribute greatly to teratoma formation. Moreover, Miura et al. discovered that the presence or absence of c-Myc in iPSCs and drug selection for NANOG or Fbxo15 expression [28, 31], all of which are considered closely associated with tumorigenesis, showed no correlation with teratoma formation. Namely, the underlying mechanism of teratoma formation is different from that of tumor, as they do not correlate with these tumor makers.

It is still unclear why undifferentiated cells remain in iPSC grafts. However, iPSC derivatives of different origins do demonstrate different teratoma-forming propensity [16, 28]. For instance, iPSCs derived from tail-tip fibroblasts showed the highest propensity for teratoma formation while iPSCs from embryonic fibroblasts and gastric epithelial cells showed the lowest. Since iPSCs from different origins exhibited distinctive features, it is possible that epigenetic memory, the residual features of somatic tissues, plays a role in teratoma formation. And due to epigenetic memory, iPSCs from certain cell lines may be likely to redifferentiate back into their initial cell type [32, 33]. Therefore, we might as well hold the belief that if we created a certain type of microenvironment supporting certain iPSCs to differentiate into NS/PCs, those derived from any other cell lines except neural ones may not be able to well differentiate and have to maintain undifferentiated status under this unfavorable condition. Besides, the inefficient methods of purifying the contaminated undifferentiated cells also aggravate the situation.

### 3.2. Substantial Tumorigenesis

Several studies have found that even if all undifferentiated cells are purged [26, 34], iPSC derivatives remain tumorigenic, as substantial tumors were present instead of teratomas. Such cases can be much worse because they are usually malignant and able to progress, invade, and metastasize. As such, understanding the mechanisms behind tumorigenesis is imperative.

The exact mechanism underlying iPSC tumorigenesis is still not clearly defined, but several factors are thought to contribute to it. Collectively, genomic and epigenomic instability correlates largely with tumorigenicity of iPSCs [35, 36]. Many factors can account for genomic instability. For instance, several oncogenes (like c-Myc and KLF4) or genes sometimes associated with tumorigenesis (like SOX2 and Oct-4) are used in the reprogramming process. Additionally, retroviral or lentiviral gene delivery systems are used in the reprogramming process and can be integrated into the genome-disrupting tumor suppressor genes and pathways. For example, the activation of transgenic Oct-4 and KLF4 has been found to induce tumor formation of NS/PCs via the Wnt/*β*-catenin signaling pathway [34]. This pathway was found to be able to enhance stabilization of telomeres, a signature of tumorigenesis, by increasing TERT expression. Furthermore, the mature cells harvested for iPSC induction have themselves already undergone multiple rounds of division and might possess their own genetic instability before induction [37]. Also, the low-efficiency reprogramming process and incomplete suppression of transgenic factors result in some partially reprogramming cells, which take part in tumor forming.

On the other hand, epigenomic instability, especially DNA methylation, also plays a role in the formation of true tumors [26]. DNA methylation has been found to have strong association with tumorigenesis in cancer tissues [38]. For instance, if oncogenes possess hypomethylation in a cell sample, such cells may show a higher likelihood to form tumors and vice versa. Consistent with this idea, 253G1-hiPSCs as well as 253G1-iPSC-NS/PCs, which had DNA hypomethylation mainly in oncogenes and hypermethylation in tumor suppressor genes, were more likely to develop tumors when compared with 207B1-hiPSCs and NS/PCs, which did not. In addition, tumorigenicity can be enhanced as induced cells are passaged because the passage of iPSCs and iPSC-derived NS/PCs further alters the epigenetic profiles via DNA methylation.

## 4. Possible Solutions to iPSC Tumorigenesis

### 4.1. Strategies to Prevent Teratoma Formation

As mentioned above, the formation of teratomas is largely attributed to undifferentiated cells. Based on this, some reports proposed various methods to address this problem including the following:

1. Increased number of passages to weaken epigenetic memory. Several studies observed the loss of epigenetic memory with increased passage number [33, 39]. iPSCs at late passage and ESCs became indistinguishable and acquired similar ability of differentiation. Therefore, the undifferentiated cell correspondingly reduced when iPSCs were capable enough of differentiation into other cells. While the underlying mechanism is not quite clear, two possible aspects may account for this phenomenon: (i) most of the iPSCs will gradually erase somatic marks as those cells passaged and/or (ii) those rare, fully reprogrammed cells become superior and then are picked up step by step [39].
2. Take advantage of epigenetic memory characteristics and use it to reprogram iPSCs away from a teratoma-inducing lineage. The propensity of iPSCs to differentiate bias into their starting cell lineage could be exploited to produce certain cell types. For example, to get more NS/PCs from iPSCs, we may ideally think of the utilization of neural cells. Some previous reports [40, 41] also confirmed that, in comparison with other cell lineages of origin, iPSCs from neural tissue are more likely and efficient to differentiate into NS/PCs. The more likely to differentiate into other cells, the less possibility of forming teratomas.
3. Improve the ability to purify iPSC-NS/PCs. It is essential to better gain bona fide iPSC-NS/PCs, as the potential for contamination with undifferentiated iPSCs presents a big chance of forming teratomas. Therefore, scientists have tried many ways to achieve the common goal including finding more specific cell surface makers and diminishing residual undifferentiated cells like inhibiting DNA topoisomerase II or stearoyl-coA desaturase [21, 42]. Accordingly, it does help but it still urgently needs to pan for desired unique makers or proper methods of depleting undifferentiated cells.
4. Transplant more mature cells instead of naive ones. It has been observed that teratomas formed from iPSC-derived NS/PCs were much smaller than those directly from iPSCs, indicating that predifferentiation of iPSCs can reduce certain aspects of tumorigenicity [43]. Consequently, grafting iPSCs directly in the treatment of SCI is not recommended.

Taken together, these ways to address undifferentiated cell contamination in iPSC-derived NS/PC transplants are, at least in part, currently effective, but it seems impossible for some of these methods to be translated into clinical application due to either the invasive operation or time-consumed culture to weaken epigenetic memory. And we had better transplanted relatively mature iPSC-derived NS/PCs instead of iPSC itself.

### 4.2. Strategies to Prevent True Tumors

As for substantial tumors, we also have several effective steps to reduce the risk including the following:

1. Change the reprogramming methods into integration-free methods. Virally induced iPSCs with genomic integrations of transcriptional factors easily cause insertional mutagenesis and result in continual expression of residual factors in iPSCs [44]. Thus, instead of using integrative vectors like retrovirus or lentivirus, we need to pursue integration-free methods, not perturbing the genome. Episomal vector and Sender virus vector were once thought to be ideal nonintegrating methods, as the former works as extrachromosomal DNA in the nucleus while the latter is a method of transgene-free induction. But as the potential spontaneous integration by episomal vector and the involvement viral particles, both are limited to clinical applications. Subsequently, Woltjen et al. discovered that piggyBac transposons could be integrated into genomes of the host so the reprogramming factors that they carried were able to express continuously and stably [45]. Furthermore, the piggyBac transposons could be cut out of the genomes completely [45]. Afterwards, the advent of DNA-free and viral-free methods like recombinant proteins, messager RNA, and mature microRNA made iPSCs stride towards clinical use despite being technically challenging or inefficient. Of note, iPSCs of the first clinical trial were generated by the nonintegrative method of reprogramming with recombinant proteins [46].
2. Avoid using transgenic factors of oncogenesis. The Yamanaka factors are competent enough to induce tumorigenesis playing important roles in the development and maintenance of cancer. It appears quite necessary to reduce reprogramming factors in order to decrease the possibility of tumor formation and hasten the clinical use. Nakagawa et al. initiated a series of experiments to test whether fewer factors are capable enough of inducing the stem cell. It was found that exogenous c-Myc was not necessarily needed to generate iPSCs [31]. They then found that exogenous Oct-4 together with KLF4 or SOX2 could produce iPSCs from NSC. Furthermore, they discovered that transcriptional factor Oct-4 alone is sufficient to acquire iPSCs [41]. Although the low-reprogramming efficiency of them limits their applications, their attempt provides us with new ideas.
3. Reduce undesirable DNA methylation. Decreasing DNA methylation of tumor suppressor genes and increasing that of oncogenes can certainly reduce the rate of tumor formation from iPSCs. The application of knocking down the maintenance methyltransferase DNMT1 or the demethylating agent like 5-AZA can reduce residual methylation of resulting cells and convert them to authentic pluripotent cells [33]. Besides, Mikkelsen et al. found that demethylation appears passage dependent [47]. Some reports showed that DNA methylation could be gradually erased as the cells were passaged [33, 39]. Iida et al. [26], however, found that DNA methylation patterns became more unstable with cells passaged. Maybe, this can be accounted for the fact that the cell clones that they used were different indicating that the ability of passaging to gradually diminish methylation cannot be applicable to all clones.
4. Establish reliable ways to distinguish the safe and unsafe cell clones. By virtue of the teratoma-forming activity of the iPSC derivatives after their transplantation [28], we are capable of differentiating the safe iPSC clones from all cultured cell clones. Preevaluated safe clones can show significant therapeutic effects without tumor formation [16–18], while preevaluated unsafe clones demonstrate high rates of tumor formation. Iida discovered that methylation states of CAT and PSMD5 genes can be applied to discriminate between safe and unsafe hiPSC-NS/PCs [26].

In brief, across the entire process of iPSC generation and NS/PC differentiation, there are steps that can be taken to reduce nonteratoma tumor formation. These strategies mentioned above just provide some possible way to circumvent the tumorigenicity, but I am afraid that there is still a long way from clinical applications.

## 5. Conclusions

Despite numerous therapeutic discoveries in the laboratory, to our knowledge, faithfully effective treatment for spinal cord injury remains unavailable. iPSC transplantation for SCI is currently an unrealistic strategy, but we have already recognized the huge potential of iPSCs for SCI because of their ability to self-renew and differentiate into various types of neural cells. In addition, iPSCs also avoid the ethical issues associated with some transplant sources and importantly can be performed in an autologous manner removing the need for immune suppression.

However, although the Takahashi group claimed that they were warranted to restart their clinical trials on iPSCs, safety concerns, especially tumorigenicity, still seriously limit considerations for clinical application, at least on SCI [48]. They once carried out the first clinical application of iPSCs in 2014, but were required to halt for some reasons. In this review, we focused on the two different routes of tumorigenicity and underlying mechanisms behind them. We also put forward some potential solutions to tumorigenesis. But in the current state, not enough is understood about underlying causes of tumor genesis from iPSC derivatives to completely elucidate the issue. More explorations and attempts need to be done in the future.

## Figures and Tables

**Table 1:** Some current studies of iPS cell transplantation in the SCI model. This table summarizes some of the experimental studies involving iPSC derivative transplantation into SCI models and its outcomes.

Reference	Grafts	SCI model	Outcomes
Tsuji et al. [16]	Mouse iPSC-derived neurospheres	Mouse contusive SCI at T-10 level	Preevaluated safe iPSC-derived cells promoted functional recovery without teratomas or other tumors, while the “unsafe” iPSC-derived cell resulted in teratoma forming and functional deterioration
Nori et al. [17]	Human iPSC-derived neurospheres	Mouse contusive SCI at T-10 level	Human iPSC-derived cells improved motor functional recovery without tumor formation but some pluripotent stem cells remained at 112d post-SCI
Kobayashi et al. [18]	Human iPSC-derived NS/PCs	Marmoset contusive SCI at C-5 level	Preevaluated safe iPSC-derived cells promoted functional recovery without tumors, whereas some undifferentiated cells were still presented after 16 weeks posttransplantation
Fujimoto et al. [20]	Human iPSC-derived neuroepithelial-like stem cells (hiPS-It-NESC)	Mouse contusive SCI at T9-10 level	hiPS-It-NESCs promote recovery of motor function and reconstruct neuronal circuity with no tumors up to 12 weeks after SCI
LiuTang et al. 2013	Human iPSC-derived NSCs	Rhesus monkey contusive SCI at T9 level	Human iPSC-derived NSCs migrated into damaged regions and showed functional recovery with no tumors after 30 days post-SCI
Lu et al. [19]	Human iPSC-NSCs	Rats and mice lateral spinal cord lesions at C5 level	Human iPSC-NSCs showed long-distance growth of human axons without obvious functional recovery
Salewski et al. 2015	Mouse iPSC-NSCs	Clip-compression spinal cord injuries at the T6 level	Wildtype-iPSC-NSCs improved neurobehavioral function while nonmyelinating Shiverer-iPSC-NSC did not
Oh et al. 2015	iPSC-NPCs from human disc	Mouse compressional SCI at T-11 level	iPSC-NPCs promoted functional and structural recovery with no tumor formation but undifferentiated cells still existed five weeks later
Pomeshchik et al. [22]	Human iPSC-NPCs	Mouse contusive SCI at T-10 level	Transplanted cells failed to improve functional recovery but no tumor formed and undifferentiated cells were not detected
Kawabata et al. 2016	Human iPSC-OPC-enriched NS/PCs	Mouse contusive SCI at T-10 level	Transplanted cells lead to robust remyelination and enhance functional recovery without tumorigenicity
Okubo et al [21]	Human iPSC-NS/PCs with *γ*-secretase inhibitor (GSI)	Mouse contusive SCI at T-10 level	GSI-treated hiPSC-NS/PCs exhibited motor functional recovery and decreased residual immature cells
Itakura et al [27]	Human-integrated iCaspase9-iPSC-NS/PCs with chemical inducers of dimerization (CIDs)	Mouse contusive SCI at T-10 level	Transplanted cells of the CID group exhibited continually functional recovery while the control groups showed functional decline due to teratomas

## Abbreviations

ESCs: Embryonic stem cells
hiPSCs: Human iPSCs
iPSCs: Induced pluripotent stem cells
NS/PC: Neural stem cell or progenitor cell
SCI: Spinal cord injury.
